# Projecting the impact of triple CFTR modulator therapy on intravenous antibiotic requirements in cystic fibrosis using patient registry data combined with treatment effects from randomised trials

**DOI:** 10.1136/thoraxjnl-2020-216265

**Published:** 2021-09-23

**Authors:** Ruth H Keogh, Rebecca Cosgriff, Eleni-Rosalina Andrinopoulou, Keith G Brownlee, Siobhán B Carr, Karla Diaz-Ordaz, Emily Granger, Nicholas P Jewell, Alex Lewin, Clemence Leyrat, Daniela K Schlüter, Maarten van Smeden, Rhonda D Szczesniak, Gary J Connett

**Affiliations:** 1 Department of Medical Statistics, London School of Hygiene & Tropical Medicine, London, UK; 2 Cystic Fibrosis Trust, London, UK; 3 Department of Biostatistics, Erasmus Medical Center, Rotterdam, The Netherlands; 4 Department of Paediatric Respiratory Medicine, Royal Brompton and Harefield NHS Foundation Trust, London, UK; 5 Department of Public Health, Policy and Systems, Institute of Population Health, University of Liverpool, Liverpool, UK; 6 Julius Center for Health Sciences and Primary Care, University Medical Center Utrecht, Utrecht University, Utrecht, The Netherlands; 7 Division of Biostatistics & Epidemiology, Cincinnati Children's Hospital Medical Center, Cincinnati, Ohio, USA; 8 Department of Pediatrics, University of Cincinnati, Cincinnati, Ohio, USA; 9 National Institute for Health Research, Southampton Respiratory Biomedical Research Centre, University Hospital Southampton NHS Foundation Trust, Southampton, UK

**Keywords:** cystic fibrosis, clinical epidemiology, respiratory infection

## Abstract

**Background:**

Cystic fibrosis (CF) is a life-threatening genetic disease, affecting around 10 500 people in the UK. Precision medicines have been developed to treat specific CF-gene mutations. The newest, elexacaftor/tezacaftor/ivacaftor (ELEX/TEZ/IVA), has been found to be highly effective in randomised controlled trials (RCTs) and became available to a large proportion of UK CF patients in 2020. Understanding the potential health economic impacts of ELEX/TEZ/IVA is vital to planning service provision.

**Methods:**

We combined observational UK CF Registry data with RCT results to project the impact of ELEX/TEZ/IVA on total days of intravenous (IV) antibiotic treatment at a population level. Registry data from 2015 to 2017 were used to develop prediction models for IV days over a 1-year period using several predictors, and to estimate 1-year population total IV days based on standards of care pre-ELEX/TEZ/IVA. We considered two approaches to imposing the impact of ELEX/TEZ/IVA on projected outcomes using effect estimates from RCTs: approach 1 based on effect estimates on FEV_1_% and approach 2 based on effect estimates on exacerbation rate.

**Results:**

ELEX/TEZ/IVA is expected to result in significant reductions in population-level requirements for IV antibiotics of 16.1% (~17 800 days) using approach 1 and 43.6% (~39 500 days) using approach 2. The two approaches require different assumptions. Increased understanding of the mechanisms through which ELEX/TEZ/IVA acts on these outcomes would enable further refinements to our projections.

**Conclusions:**

This work contributes to increased understanding of the changing healthcare needs of people with CF and illustrates how Registry data can be used in combination with RCT evidence to estimate population-level treatment impacts.

**Key messages**

**What is the key question?**

- The newest precision medicine for cystic fibrosis (CF), elexacaftor/tezacaftor/ivacaftor (ELEX/TEZ/IVA), has been found to be highly effective in randomised controlled trials (RCTs) and is becoming available to a large proportion of UK CF patients in 2020: what will its impact be on population level requirements for intravenous antibiotics?

**What is the bottom line?**

- ELEX/TEZ/IVA is expected to result in a significant reduction in the total population requirement for intravenous antibiotics of between 16.1% (~17 800 days) and 43.6% (~39 500 days).

**Why read on?**

- Discover how we combined observational UK Cystic Fibrosis Registry data with RCT results to show how treatment effect estimates translate into population-level healthcare needs, by projecting the impact of introducing ELEX/TEZ/IVA into the UK CF population on days of intravenous antibiotic treatment in hospital and at home.

## Introduction

In the UK, approximately 10 500 people have cystic fibrosis (CF), one of the most common life-threatening genetic diseases.^1^ In recent years, precision medicines called CF transmembrane conductance regulator (CFTR) modulators have been developed to treat people with CF (pwCF). They work through targeted effects on CFTR processing and function and are specific for certain CF-causing gene mutations. Online supplemental table 1 summarises the CFTR modulator treatments that have been developed and current access within the UK. The first CFTR modulator to be approved was ivacaftor in 2012. It treats pwCF with at least one copy of a gating mutation, representing <5% of the CF population. Combination treatments ivacaftor/lumacaftor (IVA/LUMAC) (‘Orkambi’) and tezacaftor/ivacaftor (TEZ/IVA) (‘Symkevi’) gained National Health Service (NHS) funding approval in autumn 2019 for a larger proportion of the population based on genotype. Elexacaftor/tezacaftor/ivacaftor (ELEX/TEZ/IVA) (‘Kaftrio’ in Europe, ‘Trikafta’ in the USA) received marketing authorisation in Europe in 2020 and began to be made available for use across the UK from mid-2020. Randomised controlled trials (RCTs) show that ELEX/TEZ/IVA is a highly effective modulator in patients with two copies of the F508del gene mutation or one copy plus another minimal function gene mutation. European licencing and commissioning guidance and approval for funding have expanded the range of pwCF in the UK able to access ELEX/TEZ/IVA to anyone over the age of 12 with at least a single copy of the F508 mutation and people carrying a single copy of a listed mutation shown to be responsive to in vitro treatment. Over 5800 pwCF in the UK are eligible for this treatment and uptake has been rapid.^2^

10.1136/thoraxjnl-2020-216265.supp1 Supplementary data

Based on RCT results, it is anticipated that introducing ELEX/TEZ/IVA will significantly improve lung function, reduce pulmonary exacerbations, intravenous (IV) antibiotic use and improve quality of life. Understanding these impacts on NHS practice, and in particular on hospital bed utilisation, is vital to planning service provision for the UK CF population versus the competing needs of the wider population given the ongoing challenges of COVID-19. As yet, the health economic impacts are unknown. In this study, we have used observational data from the UK CF Registry combined with RCT results to project the potential impact of ELEX/TEZ/IVA on reducing IV antibiotic treatment at the UK CF population level.

The study aims were (1) to develop a prediction model that can be used to estimate the number of days of IV antibiotics used by the UK CF population aged ≥12 years over a 1-year period while receiving current standards of care, and (2) to project the impact of introducing ELEX/TEZ/IVA on this outcome measure. We considered three outcomes: number of hospital bed days due to IV antibiotics (hospital-IV-days), number of days using IV antibiotics at home (home-IV-days) and their sum (combined-IV-days). For the first aim, we used UK CF Registry data from 2015 to 2017 to develop prediction models, and applied these to the most recently available data on the UK CF population recorded in 2018 to obtain estimates of population totals of each outcome over 1 year, assuming the 2018 population is approximately representative of the current population. For our second aim, we combined the predicted outcomes with evidence from RCTs on the impact of ELEX/TEZ/IVA.

## Methods

### Data

The UK CF Registry is a national, secure database sponsored and managed by the Cystic Fibrosis Trust.^3^ It records demographic and longitudinal health data on nearly all (>99%) pwCF in the UK, to date capturing over 12 000 individuals, making it a reliable resource for estimating population outcomes. Data are collected in a standardised way at annual visits and stored on a centralised database.

This study uses data from visits recorded from 2015 to 2018. We excluded visits at which individuals were aged <12 years and visits post-transplant. At each visit, the Registry records the start and end dates of IV antibiotic use episodes covering the period since the previous visit. These were used to calculate hospital-IV-days, home-IV-days and combined-IV-days for each individual in the year following visits in 2015, 2016 and 2017. Dates of IV antibiotic use between an individual’s last visit and date of death are not typically recorded.

We also used data on several covariates (see section on Prediction model development and evaluation (step 1)).

### Prediction model overview

The analysis involved the following steps, discussed in more detail below:

Step 1. Registry data from 2015 to 2017 were used to develop and evaluate prediction models for hospital-IV-days and home-IV-days over a 1-year period based on a set of predictors measured at the start of the period.

Step 2. The prediction models were applied to the 2018 patient data to estimate hospital-IV-days and home-IV-days over the following 1-year period.

Step 3. Since the most recently available data from the UK CF Registry (2018) pre-dates the general availability of TEZ/IVA and IVA/LUMAC, we also used findings from RCTs to incorporate the potential impact of TEZ/IVA on our results. The majority of people aged ≥12 and eligible for IVA/LUMAC and TEZ/IVA are using TEZ/IVA. Evidence on treatment effects from RCTs of TEZ/IVA was used to impose the potential impact of TEZ/IVA on hospital-IV-days and home-IV-days over 1 year following annual visits in 2018, for individuals in a genotype group that is now (since 2019) eligible to receive TEZ/IVA.

Step 4. Evidence on treatment effects from RCTs of ELEX/TEZ/IVA was used to impose the impact of ELEX/TEZ/IVA on hospital-IV-days and home-IV-days over 1 year following annual visits in 2018, for individuals with genotypes such that they are eligible to receive this treatment, including those who have switched from TEZ/IVA to ELEX/TEZ/IVA.

**Figure 1:**
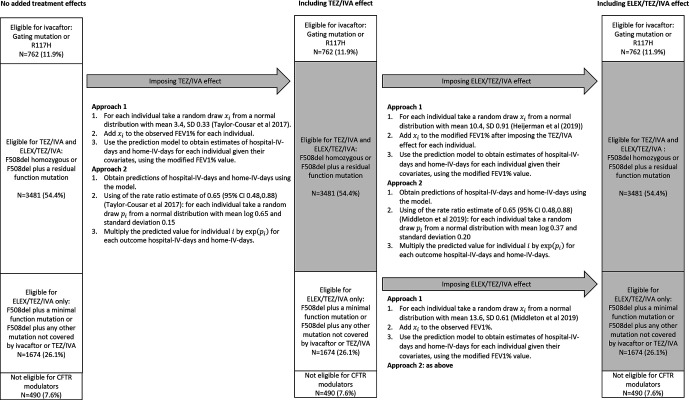
Overview of approaches to imposing the potential effect of TEZ/IVA and ELEX/TEZ/IVA on hospital-IV-days and home-IV-days. CFTR, CF transmembrane conductance regulator; ELEX, elexacaftor; IVA, ivacaftor; TEZ, tezacaftor.

Figure 1 provides a schematic overview of our analytical plan.

### Prediction model development and evaluation (step 1)

Separate prediction models were fitted for the outcomes hospital-IV-days and home-IV-days using data on individuals observed at annual review visits in 2015, 2016 and 2017 and who did not die before their next visit. The outcomes are counts of days and many individuals have counts of zero. To account for this, the analysis uses a ‘hurdle’ model: a two-part model, where the first part is a logistic model for the probability of a zero, and the second part is a zero-truncated negative binomial model for positive counts. There are other peaks in the outcome distributions, particularly at multiples of 14 days, due to IV antibiotic prescribing practices (online supplemental figure 1), and we considered extended hurdle models allowing additional peaks but these did not provide improved predictions.

The models, which were fitted using combined data from 2015 to 2017, are detailed in online supplemental section S1. Models included age, sex, and genotype and the following time-dependent predictors, which were measured at the start of each 1-year period: FEV_1_% (obtained using Global Lung Function Initiative equations^4^) and body mass index obtained as single measures on the day of the annual visit, FEV_1_% measured at the previous visit, infection with *Pseudomonas aeruginosa*, *Staphylococcus aureus* and *Burkholderia cepacia* (in the past year), diagnosis of CF-related diabetes, hospital-IV-days and home-IV-days over the past year. The included covariates were selected based on clinical consensus, and we aimed to include confounders of the associations between FEV_1_% and the outcomes, which was required for one of our approaches to imposing treatment effects in step 3 (approach 1, see below). Continuous and count covariates were modelled using splines. Genotype was categorised into six groups reflecting eligibility for CFTR modulators (table 1). There were missing data in some time-dependent covariates. Due to the relatively low missingness (online supplemental table 3), we used the last-observation-carried-forward. There remained a minimal amount of missingness and individuals with remaining missing data were excluded. There was also some missingness in the outcome, including due to missing IV antibiotic episode dates between an individual’s last visit and his/her death. Individuals with missing outcome were excluded from the prediction model development. The predicted combined-IV-days was the sum of the predicted hospital-IV-days and home-IV-days.

**Table 1:** Descriptive statistics for covariates included in the prediction model and outcome variables, excluding individuals with missing data in covariates or the outcomes, by year

		2015 (n=5929)	2016 (n=6075)	2017 (n=5963)	2018 (n=6407)
**Covariates**					
Age	Median (IQR)	25.1 (19.0–53.5)	25.8 (19.0–54.1)	26.0 (19.3–55.1)	26.4 (19.5–56.0)
Sex	Male, n (%)	3263 (53.6%)	3369 (53.7%)	3442 (53.9%)	3435 (53.6%)
	Female, n (%)	2829 (46.4%)	2903 (46.3%)	2938 (46.1%)	2972 (46.4%)
FEV_1_%	Median (IQR)	67.1 (47.6–84.7)	68.2 (48.0–84.8)	68.1 (47.8–85.4)	68.6 (47.7–85.3)
FEV_1_% previous year	Median (IQR)	69.0 (49.5–85.7)	69.5 (50.3–85.9)	70.0 (50.5–86.0)	70.2 (50.2–86.3)
Body mass index	Median (IQR)	21.7 (19.5–24.2)	21.8 (19.5–24.3)	21.8 (19.6–24.5)	21.8 (19.6–24.4)
*Pseudomonas aeruginosa*	No, n (%)	2446 (40.2%)	2817 (44.9%)	2930 (45.9%)	2916 (45.5%)
	Yes, n (%)	3646 (59.8%)	3455 (55.1%)	3450 (54.1%)	3491 (54.5%)
*Staphylococcus aureus*	No, n (%)	3567 (58.6%)	3976 (63.4%)	3937 (61.7%)	3893 (60.8%)
	Yes, n (%)	2525 (41.4%)	2296 (36.6%)	2443 (38.3%)	2514 (39.2%)
*Burkholderia cepacia*	No, n (%)	5790 (95.0%)	5956 (95.0%)	6083 (95.3%)	6101 (95.2%)
	Yes, n (%)	302 (5.0%)	316 (5.0%)	297 (4.7%)	306 (4.8%)
CF-related diabetes	No, n (%)	3764 (61.8%)	3877 (61.8%)	3941 (61.8%)	4085 (63.8%)
	Yes, n (%)	2328 (38.2%)	2395 (38.2%)	2439 (38.2%)	2322 (36.2%)
Genotype	F508del homozygous	3026 (49.7%)	3079 (49.1%)	3102 (48.6%)	3131 (48.9%)
	F508+ minimal	1095 (18.0%)	1118 (17.8%)	1112 (17.4%)	1115 (17.4%)
	F508+ residual	307 (5.0%)	321 (5.1%)	337 (5.3%)	350 (5.5%)
	F508+ other/unknown	523 (8.6%)	549 (8.8%)	564 (8.8%)	559 (8.7%)
	Any gating mutation or R117H	686 (11.3%)	721 (11.5%)	765 (12.0%)	762 (11.9%)
	Other/unknown	455 (7.5%)	484 (7.7%)	500 (7.8%)	490 (7.6%)
Hospital-IV-days, past year*	Zero, n (%)	3029 (49.7%)	3830 (61.1%)	3801 (59.6%)	3755 (58.6%)
	Median of non-zeros (IQR)	25.0 (14.0,42.0)	15.0 (9.0,32.0)	14.0 (9.0,31.0)	15.0 (9.0,32.0)
Home-IV-days, past year*	Zero, n (%)	3025 (49.7%)	4400 (70.2%)	4500 (70.5%)	4489 (70.1%)
	Median of non-zeros (IQR)	26.0 (14.0–43.0)	18.0 (13.0–33.0)	20.0 (13.0–34.0)	19.0 (13.0–34.0)
**Outcome variables**					
Hospital-IV-days,*†	Zero, n (%)	3564 (60.1%)	3571 (58.8%)	3407 (57.1%)	–
	Median of non-zeros (IQR)	15.0 (9.0–33.0)	15.0 (9.0–33.0)	16.0 (10.0–33.0)	–
Home-IV-days,*†	Zero, n (%)	4079 (68.8%)	4218 (69.4%)	4100 (68.8%)	–
	Median of non-zeros (IQR)	18.0 (13.0–34.0)	20.0 (13.0–34.0)	20.0 (13.0–35.0)	–

*When the start and end dates for a given episode were the same, the number of days was counted as 1; otherwise, the number of days for that episode was counted as the difference between the start and end dates.

†These are the counts of hospital-IV-days and home-IV-days in the year following the annual review visit in 2015, 2016 and 2017. By contrast, ‘Hospital-IV-days, past year’ and ‘Home-IV-days, past year’ are the counts in the year leading up to the annual review visit.

The predictive performance of the model was evaluated using discrimination and calibration measures. Overfitting-corrected estimates of these measures were obtained using a bootstrapping approach.^5^ The model was fitted in each bootstrap sample and evaluated in the same sample (in-sample performance) and in the subset of individuals not in the bootstrap sample (out-of-sample performance). Assessment measures were obtained in-sample and out-of-sample, and results are based on means across 1000 bootstrap samples. Discrimination was assessed on the part of the model that predicts whether an individual’s outcome is zero using the area under the receiver operating characteristic (ROC) curve. We assessed overall predictive performance and calibration through the bias and root mean squared error of the predicted counts. Model calibration was also assessed graphically—we divided the predicted outcomes into 100 ordered groups of equal size (based on quantiles) and compared the mean observed outcome with the mean predicted outcome in each group. Calibration was a key consideration in this investigation because we used the model to obtain predicted counts in subsets of the population under different potential treatment effects.^6^

### Estimation of population totals (step 2)

We used the prediction model fitted to the complete 2015–2017 data to obtain predictions of hospital-IV-days, home-IV-days and combined-IV-days for each individual in the 2018 data, for which observed outcomes were not available. We present the population totals and population means for each outcome for the whole 2018 CF population and within groups defined by access to CFTR modulators. Estimates are accompanied by 95% prediction intervals (95% PIs) (online supplemental section S2).

### Imposing the potential impact of TEZ/IVA and ELEX/TEZ/IVA using RCT results (steps 3 and 4)

There have been two phase III RCTs of TEZ/IVA and two of ELEX/TEZ/IVA in pwCF aged ≥12 years (online supplemental table 2).^7–10^ To impose the effects of TEZ/IVA, we used results from the study of Taylor-Cousar *et al* 7 comparing TEZ/IVA with placebo in F508del homozygotes. The primary endpoint was increase in FEV_1_% at 24 weeks, and the increase in the TEZ/IVA group was 3.4 points of FEV_1_% (95% CI 2.7 to 4.0). Despite the modest impact on FEV_1_%, the rate ratio for the second outcome of number of pulmonary exacerbations at 24 weeks was 0.65 (95% CI 0.48 to 0.88). Middleton *et al* 9 investigated the ELEX/TEZ/IVA impact in F508del heterozygotes with a minimal function mutation. The estimated increase in FEV_1_% at 4 weeks (primary outcome) in the ELEX/TEZ/IVA group was 13.6 (95% CI 12.4 to 14.8). The rate ratio for exacerbations (leading to hospitalisation or treatment with IV antibiotics) up to 24 weeks (secondary outcome) was 0.37 (95% CI 0.25 to 0.55). Heijerman *et al* 10 investigated the ELEX/TEZ/IVA impact in F508del homozygotes, with TEZ/IVA as the comparator. The estimated increase in FEV_1_% at 4 weeks in the ELEX/TEZ/IVA group was 10.4 (95% CI 8.6 to 12.2).

**Figure 2:**
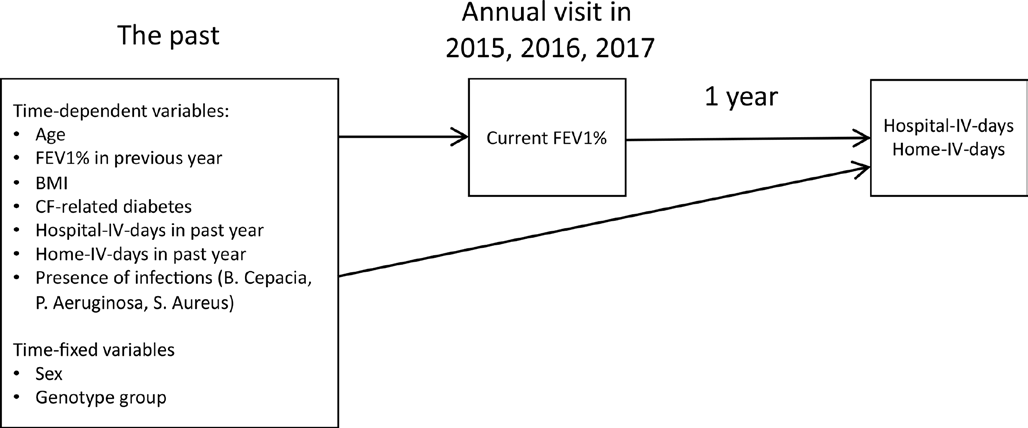
Directed acyclic graph showing assumed relationships between covariates and outcomes for approach 1. BMI, body mass index; CF, cystic fibrosis; IV, intravenous.

We considered two approaches to imposing the impact of TEZ/IVA and ELEX/TEZ/IVA on the outcomes, which require different assumptions: approach 1—using RCT results on the impact of the treatments on FEV_1_%; approach 2—using RCT results on the impact of the treatments on pulmonary exacerbation rate. Approach 1 assumes the treatment effect on hospital-IV-days and home-IV-days is mediated entirely through its effect on FEV_1_% (see figure 2). Under approach 2, we make the assumption that the rate ratio for the treatment effect on exacerbation rate can be applied directly to rates of hospital-IV-days and home-IV-days —that is, we assume approximate equivalence between exacerbations and requirement for IV antibiotics both in hospital and at home. The two approaches therefore provide complementary evidence and have different merits. Individuals who were F508del homozygous or F508del heterozygous with a residual function mutation were considered eligible for TEZ/IVA and were assumed to be using it. These individuals were also assumed eligible for ELEX/TEZ/IVA, alongside F508del individuals heterozygous for minimal function gene mutations, but excluding those eligible for ivacaftor.

Figure 1 details the two approaches. When imposing the treatment effects from the RCTs, we accounted for the uncertainty in the effect estimates and we obtained 95% PIs for population total outcomes (see online supplemental section S2).

For approach 1, the expected impacts of TEZ/IVA and ELEX/TEZ/IVA are imposed, in turn, on each eligible individual’s observed FEV_1_% value in the 2018 data, and the prediction model is then used to obtain predicted outcomes using the modified FEV_1_% values. To impose the potential effect of TEZ/IVA and ELEX/TEZ/IVA on FEV_1_% in our population using the RCT results, the regression coefficient(s) for FEV_1_% in the prediction model should have an interpretation as an approximation of the causal effect of FEV_1_% on the outcome. The prediction model should therefore include FEV_1_% plus confounders of the association between FEV_1_% and the outcome. The set of predictors included in the model are all temporally prior to the FEV_1_% measure and were selected as potential confounders, as well as our expectation that they would be predictors of the outcomes (figure 2).

For approach 2, we used the prediction models to obtain predicted hospital-IV-days and home-IV-days for each individual and then reduced these by a percentage determined by the RCT estimates of the effects of TEZ/IVA and ELEX/TEZ/IVA on exacerbation rates.

## Results

### Descriptive statistics

Between 2015 and 2018, 7461 individuals aged ≥12 years had data recorded in the Registry at least once, after excluding individuals post-transplant. Prediction models were developed using data from 2015 to 2017. After exclusions due to missing data (see online supplemental table 3), among which 4% were excluded because they had missing outcome data due to death, prediction model development was based on 17 967 annual data records on 6731 individuals, whose characteristics are summarised in table 1. In the combined 2015–2017 data, 58.7% of hospital-IV-days outcomes are zero, 69% of home-IV-days are zero and 48% had no IV antibiotic days either at hospital or at home.

### Model development and evaluation

**Figure 3:**
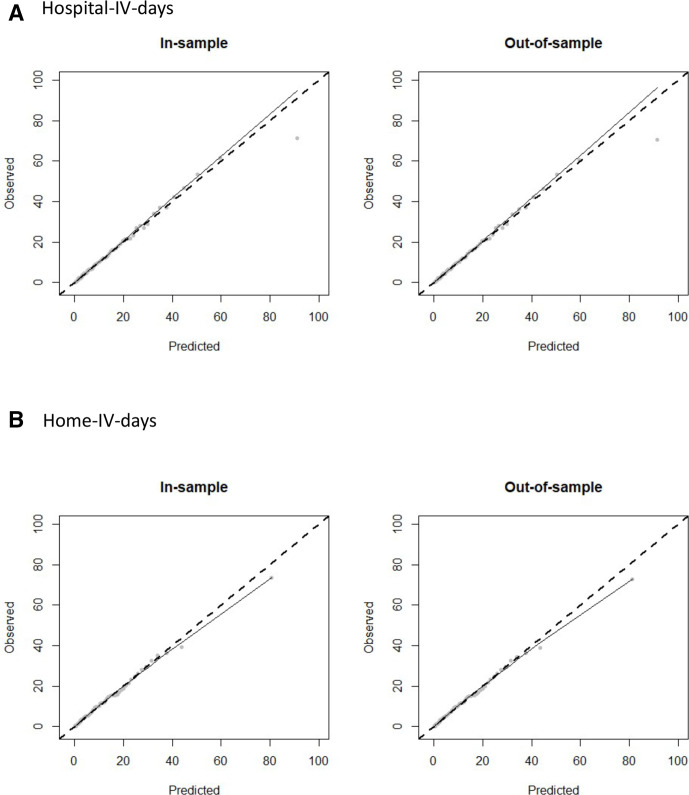
Plots showing the mean observed outcome in groups defined by 100ths of the distribution of the predicted outcome, against the mean predicted outcome in each group. Each point was obtained as the average over 1000 bootstrap samples. The solid line is the lowess curve. The dashed line is the line of equality. IV, intravenous.

Parameter estimates from the prediction models are shown in online supplemental table 4. Table 2 and figure 3 show the results from assessing the predictive performance. Discrimination was assessed on the part of the model that predicts whether an individual’s outcome is zero. The AUC was 0.81 for hospital-IV-days and 0.82 for home-IV-days, indicating reasonable performance at discriminating between individuals who have a zero and non-zero count (a value of 0.5 would indicate that the model performed no better than chance and a value of 1 would indicate perfect discrimination). The out-of-sample values were only slightly lower than the in-sample values. Looking at the overall model performance, bias was close to 0, and correspondingly, the observed population totals were close to the predicted totals. The root mean squared errors were similar for the two outcomes and indicate a reasonably substantial amount of variation between the observed and predicted outcomes.^11^ Such variability is reflected in the prediction intervals when the model is applied to the 2018 data. Calibration plots (figure 3) show that the models are well calibrated, meaning that the observed outcomes are, on average, similar to the predicted outcomes.

**Table 2:** Model evaluation results based on averages over 1000 bootstrap samples

	In-sample	Out-of-sample
Hospital-IV-days		
AUC for the probability of a zero count	0.809 (0.802 to 0.815)	0.807 (0.798 to 0.815)
Bias (days)	0.089 (0.041 to 0.133)	0.078 (−0.447 to 0.650)
RMSE (days)	17.13 (16.34 to 17.87)	17.18 (16.26 to 18.53)
Observed total (days)	191 143 (185 875 to 196 754)	70 325 (67 495 to 73 321)
Predicted total (days)	192 745 (187 173 to 198 218)	70 841 (68 249 to 73 514)
Home-IV-days		
AUC for the probability of a zero count	0.822 (0.815 to 0.828)	0.820 (0.811 to 0.828)
Bias (days)	0.092 (0.038 to 0.151)	0.063 (–0.424 to 0.589)
RMSE (days)	15.83 (14.51 to 17.85)	15.92 (14.40 to 19.78)
Observed total (days)	149 940 (144 790 to 154 897)	55 215 (52 810 to 57 748)
Predicted total (days)	151 585 (146 256 to 156 826)	55 627 (53 320 to 58 318)

The model fitted in each bootstrap sample was evaluated in-sample and out-of-sample. Estimated 95% CI are given in parentheses and were obtained using the 2.5th and 97.5th percentiles across the 1000 bootstrap samples.

AUC, area under the curve; RMSE, root mean squared error.

### Predicted population totals and the potential impact of TEZ/IVA and ELEX/TEZ/IVA

The 2018 data include 6407 individuals aged ≥12 years. Of these individuals, 54.3% (n=3481) were eligible for both TEZ/IVA and ELEX/TEZ/IVA, and a further 26.1% (n=1674) were only eligible for ELEX/TEZ/IVA according to the definition of eligibility used in this paper. Table 3 shows the predicted population totals for each outcome in the 1 year following the 2018 visit and table 4 shows the predicted population means. Before imposing any treatment effects, the predicted population totals are 67 700 (95% PI 64 700 – 71 300) hospital-IV-days and 47 300 (95% PI 44 800 – 49 900) home-IV-days. When imposing the potential impact of TEZ/IVA and ELEX/TEZ/IVA on the population totals, we obtained somewhat different results between approaches 1 and 2, with the population totals of hospital-IV-days, home-IV-days and combined-IV-days being considerably smaller using approach 2, thus suggesting a larger treatment effect.

**Table 3:** Estimated population totals for each outcome in 1 year following the 2018 visit, presented as N/1000 (95% prediction interval (95% PI)), and % reductions: with no treatment effects applied, with the effect of TEZ/IVA imposed and with the effect of ELEX/TEZ/IVA imposed

Eligibility group	No treatment effects	With TEZ/IVA effect applied to eligible individuals		With ELEX/TEZ/IVA effect applied to eligible individuals, including those assumed to switch from TEZ/IVA	
	N/1000 (95% PI)	N/1000 (95% PI)	% reduction (95% PI)	N/1000 (95% PI)	% reduction (95% PI)
**(A) Using approach 1 for imposing treatment effects**					
Hospital-IV-days					
Full cohort*	67.7 (64.7, 71.3)	64.5 (61.3, 67.7)	4.8 (–1.2, 10.1)	51.6 (48.5, 54.7)	20.0 (14.3, 25.2)
TEZ/IVA+ELEX/TEZ/IVA†	41.5 (39.1, 44.4)	38.3 (36.0, 40.8)	7.7 (0.5, 14.4)	30.3 (28.0, 32.7)	20.8 (13.5, 27.4)
ELEX/TEZ/IVA‡	60.2 (57.3, 63.6)	57.0 (54.0, 60.1)	5.3 (–0.8, 11.2)	44.0 (41.0, 47.0)	22.7 (16.7, 28.5)
Home-IV-days					
Full cohort	47.3 (44.8, 49.9)	46.1 (43.7, 48.9)	2.5 (–4.2, 8.3)	41.3 (38.9, 44.1)	10.2 (3.7, 16.4)
TEZ/IVA+ELEX/TEZ/IVA	30.1 (28.2, 32.2)	29.0 (27.0, 31.3)	3.9 (–4.5, 11.7)	25.9 (24.1, 28.1)	10.2 (2.0, 18.1)
ELEX/TEZ/IVA	42.7 (40.4, 45.1)	41.5 (39.1, 44.2)	2.7 (–4.5, 9.3)	36.7 (34.4, 39.3)	11.4 (4.3, 17.9)
Combined-IV-days					
Full cohort	115.0 (110.9, 119.5)	110.6 (106.7, 114.9)	3.8 (–0.4, 8.0)	92.8 (92.8, 92.8)	16.1 (13.0, 19.2)
TEZ/IVA+ELEX/TEZ/IVA	71.7 (68.5, 75.2)	67.3 (64.1, 70.6)	6.1 (0.7, 11.4)	56.3 (56.3, 56.3)	16.4 (12.2, 20.3)
ELEX/TEZ/IVA	102.9 (99.0, 107.3)	98.5 (94.9, 102.7)	4.3 (–0.4, 8.6)	80.7 (80.7, 80.7)	18.1 (14.9, 21.4)
**(B) Using approach 2 for imposing treatment effects**					
Hospital-IV-days					
Full cohort*	67.7 (64.6, 71.2)	53.5 (50.9, 56.3)	21.0 (20.0, 21.9)	30.3 (28.7, 32.0)	43.4 (42.2, 44.6)
TEZ/IVA+ELEX/TEZ/IVA†	41.5 (39.2, 44.3)	27.3 (25.7, 29.2)	34.3 (33.5, 35.1)	15.7 (14.7, 16.7)	42.6 (41.4, 43.6)
ELEX/TEZ/IVA‡	60.2 (57.1, 63.5)	46.0 (43.6, 48.6)	23.7 (22.7, 24.6)	22.7 (21.5, 24.0)	50.6 (49.7, 51.5)
Home-IV-days					
Full cohort	47.3 (44.9, 49.9)	36.9 (35.1, 39.1)	21.8 (20.8, 22.9)	20.7 (19.5, 22.0)	44.0 (42.6, 45.3)
TEZ/IVA+ELEX/TEZ/IVA	30.1 (28.2, 32.2)	19.8 (18.4, 21.3)	34.3 (33.3, 35.2)	11.4 (10.6, 12.3)	42.6 (41.2, 43.8)
ELEX/TEZ/IVA	42.7 (40.3, 45.2)	32.4 (30.5, 34.3)	24.2 (23.1, 25.2)	16.1 (15.2, 17.1)	50.2 (49.2, 51.3)
Combined-IV-days					
Full cohort	115.0 (111.0, 119.4)	90.5 (87.2, 94.0)	21.3 (20.6, 22.0)	51.0 (49.0, 53.1)	43.6 (42.7, 44.6)
TEZ/IVA+ELEX/TEZ/IVA	71.7 (68.6, 75.1)	47.1 (45.0, 49.5)	34.3 (33.6, 34.9)	27.1 (25.8, 28.5)	42.6 (41.7, 43.5)
ELEX/TEZ/IVA	102.9 (98.8, 107.3)	78.3 (75.2, 81.6)	23.9 (23.1, 24.6)	38.8 (37.3, 40.5)	50.4 (49.7, 51.1)

For the TEZ/IVA effect results, the % reduction is relative to the situation with no treatment effects. For the ELEX/TEZ/IVA results, the % reduction is relative to the TEZ/IVA results.

*The full cohort of N=6407 individuals.

†The subset eligible for both TEZ/IVA and ELEX/TEZ/IVA (n=3481).

‡The whole set of individuals eligible for ELEX/TEZ/IVA, including those assumed to switch from TEZ/IVA (n=5155).

ELEX, elexacaftor; IVA, ivacaftor; TEZ, tezacaftor.

**Table 4:** Estimated population means for each outcome in 1 year following the 2018 visit, presented as N/1000 (95% prediction interval (95% PI)), and % reductions: with no treatment effects applied, with the effect of TEZ/IVA imposed and with the effect of ELEX/TEZ/IVA imposed

Eligibility group	No treatment effects	With TEZ/IVA effect applied to eligible individuals		With ELEX/TEZ/IVA effect applied to eligible individuals, including those assumed to switch from TEZ/IVA	
	Mean (95% PI)	Mean (95% PI)	% reduction (95% PI)	Mean (95% PI)	% reduction (95% PI)
**(A) Using approach 1 for imposing treatment effects**					
Hospital-IV-days					
Full cohort*	10.6 (10.1, 11.1)	10.1 (9.6, 10.6)	4.8 (–1.2, 10.1)	8.0 (7.6, 8.5)	20.0 (14.3, 25.2)
TEZ/IVA+ELEX/TEZ/IVA†	11.9 (11.2, 12.8)	11.0 (10.4, 11.7)	7.7 (0.5, 14.4)	8.7 (8.0, 9.4)	20.8 (13.5, 27.4)
ELEX/TEZ/IVA‡	11.7 (11.1, 12.3)	11.1 (10.5, 11.7)	5.3 (–0.8, 1.2)	8.5 (8.0, 9.1)	22.7 (16.7, 28.5)
Home-IV-days					
Full cohort	7.4 (7.0, 7.8)	7.2 (6.8, 7.6)	2.5 (–4.2, 8.3)	6.4 (6.1, 6.9)	10.2 (3.7, 16.4)
TEZ/IVA+ELEX/TEZ/IVA	8.7 (8.1, 9.2)	8.3 (7.7, 9.0)	3.9 (–4.5, 11.7)	7.5 (6.9, 8.1)	10.2 (2.0, 8.1)
ELEX/TEZ/IVA	8.3 (7.8, 8.8)	8.0 (7.6, 8.6)	2.7 (–4.5, 9.3)	7.1 (6.7, 7.6)	11.4 (4.3, 17.9)
Combined-IV-days					
Full cohort	18.0 (17.3, 18.6)	17.3 (16.7, 17.9)	3.8 (–0.4, 8.0)	14.5 (14.5, 14.5)	16.1 (13.0, 19.2)
TEZ/IVA+ELEX/TEZ/IVA	20.6 (19.7, 21.6)	19.3 (18.4, 20.3)	6.1 (0.7, 11.4)	16.2 (16.2, 16.2)	16.4 (12.2, 20.3)
ELEX/TEZ/IVA	20.0 (19.2, 20.8)	19.1 (18.4, 19.9)	4.3 (–0.4, 8.6)	15.7 (15.7, 15.7)	18.1 (14.9, 21.4)
**(B) Using approach 2 for imposing treatment effects**					
Hospital-IV-days					
Full cohort*	10.6 (10.1, 11.1)	8.4 (7.9, 8.8)	21.0 (20.0, 21.9)	4.7 (4.5, 5.0)	43.4 (42.2, 44.6)
TEZ/IVA+ELEX/TEZ/IVA†	11.9 (11.3, 12.7)	7.8 (7.4, 8.4)	34.3 (33.5, 35.1)	4.5 (4.2, 4.8)	42.6 (41.4, 43.6)
ELEX/TEZ/IVA‡	11.7 (11.1, 12.3)	8.9 (8.5, 9.4)	23.7 (22.7, 24.6)	4.4 (4.2, 4.7)	50.6 (49.7, 51.5)
Home-IV-days					
Full cohort	7.4 (7.0, 7.8)	5.8 (5.5, 6.1)	21.8 (20.8, 22.9)	3.2 (3.0, 3.4)	44.0 (42.6, 45.3)
TEZ/IVA+ELEX/TEZ/IVA	8.7 (8.1, 9.3)	5.7 (5.3, 6.1)	34.3 (33.3, 35.2)	3.3 (3.0, 3.5)	42.6 (41.2, 43.8)
ELEX/TEZ/IVA	8.3 (7.8, 8.8)	6.3 (5.9, 6.7)	24.2 (23.1, 25.2)	3.1 (2.9, 3.3)	50.2 (49.2, 51.3)
Combined-IV-days					
Full cohort	18.0 (17.3, 18.6)	14.1 (13.6, 14.7)	21.3 (20.6, 22.0)	8.0 (7.6, 8.3)	43.6 (42.7, 44.6)
TEZ/IVA+ELEX/TEZ/IVA	20.6 (19.7, 21.6)	13.5 (12.9, 14.2)	34.3 (33.6, 34.9)	7.8 (7.4, 8.2)	42.6 (41.7, 43.5)
ELEX/TEZ/IVA	20.0 (19.2, 20.8)	15.2 (14.6, 15.8)	23.9 (23.1, 24.6)	7.5 (7.2, 7.9)	50.4 (49.7, 51.1)

For the TEZ/IVA effect results, the % reduction is relative to the situation with no treatment effects. For the ELEX/TEZ/IVA results, the % reduction is relative to the TEZ/IVA results.

*The full cohort of n=6407 individuals.

†The subset eligible for both TEZ/IVA and ELEX/TEZ/IVA (n=3481).

‡The whole set of individuals eligible for ELEX/TEZ/IVA, including those assumed to switch from TEZ/IVA (n=5155).

ELEX, elexacaftor; IVA, ivacaft; TEZ, tezacaftor.

Under approach 1 for hospital-IV-days, imposing the TEZ/IVA effect on eligible individuals indicated a 4.8% reduction (95% PI −1.2%–10.1%) in the population total (to 64 500 days (95% PI 61 300–67 700)), and imposing the ELEX/TEZ/IVA effect (including for those assumed to switch from TEZ/IVA) indicated a further 20% reduction (95% PI 14.4%–25.2%) in the population total (to 51 600 days (95% PI 48 500–54 700)). The population total includes hospital-IV-days and home-IV-days for individuals for whom no treatment effects are applied as they are not eligible for TEZ/IVA or ELEX/TEZ/IVA. This 20% reduction in population total hospital-IV-days corresponds to a reduction in the predicted mean number of days over 1 year from 10.1 days (95% PI 9.6–10.6) to 8.0 days (95% PI 7.6–8.5). For the subset of individuals eligible for ELEX/TEZ/IVA, we estimated a reduction in the total hospital-IV-days from 57 000 (95% PI 54 000–60 100) to 44 000 (95% PI 41 000–47000) after initiating or switching to ELEX/TEZ/IVA, representing a 22.7% reduction (95% PI 16.7%–28.5%). Under approach 1, the treatment impacts are less for home-IV-days, reflecting a weaker association between FEV_1_% and home-IV-days in the prediction model. Imposing the TEZ/IVA effect indicated a 2.5% reduction (95% PI −4.2%–8.3%) in the population total home-IV-days, and imposing the ELEX/TEZ/IVA effect a further 10.2% reduction (95% PI 2.0%–18.1%). This corresponds to a reduction in the mean home-IV-days from 7.2 days (95% PI 6.8–7.6) to 6.4 days (95% PI 6.1–6.9)).

Under approach 2 for hospital-IV-days, imposing the TEZ/IVA effect gave a 21% reduction (95% PI 20.0%–21.9%) in the population total hospital-IV-days (to 53 500 days (95% PI 50 900–56 300)), and imposing the ELEX/TEZ/IVA effect (including for those assumed to switch from TEZ/IVA) a further 43.4% reduction (95% PI 42.2%–44.6%) (to 30 300 days (95% PI 28 700–32 000)). The latter corresponds to a reduction in mean hospital-IV-days from 8.4 days (95% PI 7.9–8.8) to 4.7 days. Under approach 2, the reductions for home-IV-days are very similar.

Overall, our results suggest that introducing ELEX/TEZ/IVA is expected to result in a reduction in the population total requirement for IV antibotics (combined-IV-days) of between 16.1% (approach 1) and 43.6% (approach 2), over and above the impacts of TEZ/IVA. Reasons for differences in the predicted outcomes under the two approaches are discussed below.

Our primary aim has been to provide projections of population totals, rather than individual-level predictions. However, to provide clinical context, we obtained predictions from the model for example (hypothetical) individuals under standard care pre-ELEX/TEZ/IVA and after imposing the RCT treatment effect estimates. Table 5 provides predicted number of hospital-IV-days and home-IV-days for example individuals.

**Table 5:** Predicted number of hospital and home IV antibiotic days in the next year for example patients

(A) Sets of patient characteristics A, B, C. All are aged 30 and female.			
Predictor	A	B	C
FEV_1_%	40	60	80
FEV_1_% previous year	40	60	80
Body mass index	20	22	25
*Pseudomonas aeruginosa*	Yes	Yes	No
*Staphylococcus aureus*	Yes	Yes	No
*Burkholderia cepacia*	Yes	Yes	No
CF-related diabetes	Yes	Yes	No
Hospital-IV-days, past year	28	14	0
Home-IV-days, past year	28	14	0

(B) Predicted number of hospital and home IV antibiotic days in the next year for nine example patients under no treatment effects, and imposing the effects of TEZ/IVA and ELEX/TEZ/IVA using approaches 1 and 2. Each set of characteristics A, B, C is considered in combination with each genotype category (1), (2), (3).										
		Genotype (1): F508del homoz (eligible for TEZ/IVA and ELEX/TEZ/IVA)			Genotype (2): F508del heteroz+minimal (eligible for ELEX/TEZ/IVA)			Genotype (3): Gating (eligible for IVA)		
		No treatment effects	+TEZ/IVA*	+ELEX/TEZ/IVA+ TEZ/IVA†	No treatment effects	+TEZ/IVA*	+ELEX/TEZ/IVA+ TEZ/IVA†	No treatment effects	+TEZ/IVA*	+ELEX/TEZ/IVA+ TEZ/IVA†
**Hospital-IV-days**										
Approach 1	A	34.2	31.8	25.3	34.6	–	25.6	28.6	–	–
	B	14.5	13.4	10.5	14.7	–	10.6	11.0	–	–
	C	1.8	1.6	1.2	1.8	–	1.2	1.2	–	–
Approach 2	A	34.2	22.2	12.7	34.6	–	12.8	28.6	–	–
	B	14.5	9.4	5.4	14.7	–	5.4	11.0	–	–
	C	1.8	1.2	0.7	1.8	–	0.7	1.2	–	–
**Home-IV-days**										
Approach 1	A	25.2	24.1	21.1	24.0	–	20.1	18.1	–	–
	B	13.2	12.7	11.4	12.5	–	10.7	8.7	–	–
	C	5.2	5.1	4.7	4.9	–	4.4	3.2	–	–
Approach 2	A	25.2	16.4	9.3	24.0	–	8.9	18.1	–	–
	B	13.2	8.6	4.9	12.5	–	4.6	8.7	–	–
	C	5.2	3.4	1.9	4.9	–	1.8	3.2	–	–

Values of model predictors were chosen for nine example patients defined by three sets of patient characteristics (A, B, C), each combined with three different genotypes: (1) F508del homozygous (eligible for TEZ/IVA and ELEX/TEZ/IVA), (2) F508del heterozygous with a minimal function mutation (eligible for ELEX/TEZ/IVA only), (3) any gating mutation (assumed to be using ivacaftor, but not eligible for TEZ/IVA or ELEX/TEZ/IVA).

*With TEZ/IVA effect applied to eligible individuals.

†With ELEX/TEZ/IVA effect applied to eligible individuals, including those assumed to switch from TEZ/IVA.

ELEX, elexacaftor; IVA, ivacaftor; TEZ, tezacaftor.

## Discussion

With the recent agreement that the NHS in England, Scotland, Wales and Northern Ireland will fund ELEX/TEZ/IVA after receiving its European license, the CF community is looking towards future planning for the changing healthcare needs of pwCF. We developed novel approaches to anticipate the impact of ELEX/TEZ/IVA on health service utilisation in the form of IV antibiotic usage. We estimated how many days of IV antibiotic treatment in hospital and at home will be required by pwCF aged ≥12 years, and how this might change following the introduction of ELEX/TEZ/IVA. In previous work we have forecasted future patient numbers,^12^ which highlighted the need to plan for a larger adult CF population. This study increases our understanding of the future needs of this population and illustrates how combining registry and RCT data can enable estimation of population level treatment impacts.

A key strength of our study is the use of the UK CF Registry, which has almost complete coverage of the UK population. Our prediction models for hospital-IV-days and home-IV-days were well calibrated and produced unbiased internally valid predictions. We evaluated two approaches to imposing the potential impacts of TEZ/IVA and ELEX/TEZ/IVA based on primary and secondary RCT outcomes, and estimated that the introduction of ELEX/TEZ/IVA is expected to result in a significant reduction in the total population requirement for IV antibiotics (combined-IV-days) of 16.1% using approach 1 (from ~110 600 to~92 800 days) and 43.6% using approach 2 (from ~90 500 to~43 600 days). These reductions were in addition to estimated benefits derived from starting TEZ/IVA within its licenced indication.

The advantage of approach 1 is that it is based on primary outcome RCT data. It assumes that the treatment effects on IV antibiotic days are mediated entirely through their effect on FEV_1_%, and that our model accurately captures these causal effects through adjustments for potential confounders. When considering what predictor variables to include in the model, we had to consider the fact that we then wanted to use the model to obtain predictions under modified values for FEV_1_%. Therefore, our considerations for the predictor variables to include were different from a standard prediction context (where the sole aim is to achieve good predictive performance)—we needed the coefficient for FEV_1_% in the prediction model to have a causal interpretation in order to apply approach 1. This approach might underestimate the effect of ELEX/TEZ/IVA on IV antibiotic days if the treatment has effects on reducing IV antibiotic days that are not mediated directly via FEV_1_%, or that are not captured by considering FEV1% measured on a single occasion. While we adjusted for past FEV_1_%, on the basis that it is likely to be a confounder, the high correlation between past and baseline FEV_1_% could also have resulted in the baseline FEV_1_% effect being underestimated. Increased understanding of the mechanisms through which CFTR modulators impact on pulmonary exacerbation rates would provide information about as yet incompletely understood wider treatment benefits that might positively impact on needs for IV antibiotics, and would enable further refinements to be made to our projections. Secondary analyses of RCT data could be used to investigate the extent to which the effect of treatment on exacerbations is mediated through its effect on FEV_1_%.

Approach 2 imposes treatment effects based on secondary RCT outcomes assuming that the rate ratio for the treatment effect on pulmonary exacerbations can be applied to rates of hospital-IV-days and home-IV-days. This assumes that each exacerbation results in approximately the same number of hospital-IV-days and home-IV-days. The RCT of Taylor-Cousar *et al* 7 considered exacerbations that led to hospitalisation or treatment with IV antibiotics, and the RCT of Middleton *et al* considered all exacerbations, and also looked separately at those resulting in hospitalisation and those requiring treatment with IV antibiotics—in this study, we used their results for all exacerbations. The rate ratios for exacerbations requiring treatment with antibiotics were even lower, suggesting that the impact on hospital-IV-days could be even greater than we have projected. A limitation of our approach is that there is no RCT evidence on the exacerbation rate ratio for ELEX/TEZ/IVA versus TEZ/IVA, which may have resulted in an overestimation of the ELEX/TEZ/IVA impact using this approach. For approach 2, the estimate of the population total number of IV antibiotic days before imposing treatment effects could have been taken from an observed population total (which does not require knowledge of any covariates), rather than estimating the total from a prediction model. However, we did not observe population total numbers of IV antibiotic days for 2018 (because these data are obtained retrospectively each year), and therefore preferred to use the prediction model to estimate the baseline population totals. This ensures consistency between approaches 1 and 2 in terms of the baseline population totals before the treatment effects are imposed.

Study limitations included not having outcome data between date of last visit and date of death for some individuals (~4%). Requirements for IV antibiotics may be increased in the months prior to death which might have resulted in a slight underestimate of population totals. Data from RCTs were restricted to pwCF with FEV_1_% between 40 and 90, whereas we considered the entire CF population and did not consider whether there could be differences in efficacy among those outside of these lung function parameters. Furthermore, the range of mutations eligible for access to ELEX/TEZ/IVA is broader than in the RCTs. Although recent data suggest a high rate of adherence to CFTR modulator treatment, decreasing adherence in the long term, outside of RCTs, might negatively impact on the efficacy of CFTR modulator treatment in real-world settings.^13^

As ELEX/TEZ/IVA has begun to be prescribed across the UK, the UK CF Registry is collecting follow-up data for all consenting pwCF prescribed this treatment. Over time, predicted outcomes from our models can be compared with real-world findings. It may be difficult to use real-world data for 2020 and 2021 to establish the impact of modulator therapies on IV antibiotic use as COVID-19 has reshaped the CF landscape with a short-term and variable reduction in overall need for antibiotics, probably as a result of decreased rates of transmissible infections during shielding. The long-term impacts of COVID-19 on CF care are unknown but are unlikely to significantly impact on long-term need for IV antibiotics. The benefits of ELEX/TEZ/IVA at a CF population level are likely to be greater in the future given the potential to extend its use to post-transplant, younger age groups and a wider range of CF genotypes. It would be of interest to perform similar analyses to predict the impact of ivacaftor on the same outcomes, using historical Registry data from before its introduction combined with RCT data. Projections from this modelling could be compared with real-world outcomes after the introduction of ivacaftor. This will enable evaluation of the accuracy of our predictions and provide further evidence for the validity of using Registry data combined with RCTs for healthcare planning.

## Data Availability

Data may be obtained from a third party and are not publicly available. This work used anonymised data from the UK Cystic Fibrosis Registry, which has Research Ethics Approval (REC Ref: 07/Q0104/2). The use of the data was approved by the Registry Research Committee (Data Request Reference 382). Data are available following application to the Registry Research Committee (https://www.cysticfibrosis.org.uk/the-work-we-do/uk-cf-registry/apply-for-data-from-the-uk-cf-registry).

## References

[1] Cystic Fibrosis Trust . Cystic fibrosis Registry report 2018, 2019. Available: https://www.cysticfibrosis.org.uk/the-work-we-do/uk-cf-registry/reporting-and-resources

[2] Vertex Pharmaceuticals Incorporated . VX-445/TEZ/IVA expanded access program for cystic fibrosis (CF) patients heterozygous for F508del mutation and a minimal function mutation (F/MF genotypes), 2019. Available: https://clinicaltrials.gov/ct2/show/NCT04058210

[3] Taylor-Robinson D , Archangelidi O , Carr SB , et al . Data resource profile: the UK cystic fibrosis registry. Int J Epidemiol 2018;47:9–10. 10.1093/ije/dyx196 29040601 PMC5837577

[4] Quanjer PH , Stanojevic S , Cole TJ , et al . Multi-ethnic reference values for spirometry for the 3-95-yr age range: the global lung function 2012 equations. Eur Respir J 2012;40:1324–43. 10.1183/09031936.00080312 22743675 PMC3786581

[5] Steyerberg EW . Clinical prediction models: a practical approach to development, validation, and updating. Second Edition. Springer, 2019.

[6] Van Calster B , McLernon DJ , van Smeden M , et al . Calibration: the Achilles heel of predictive analytics. BMC Med 2019;17:230. 10.1186/s12916-019-1466-7 31842878 PMC6912996

[7] Taylor-Cousar JL , Munck A , McKone EF , et al . Tezacaftor-Ivacaftor in patients with cystic fibrosis homozygous for Phe508del. N Engl J Med 2017;377:2013–23. 10.1056/NEJMoa1709846 29099344

[8] Rowe SM , Daines C , Ringshausen FC , et al . Tezacaftor-Ivacaftor in Residual-Function heterozygotes with cystic fibrosis. N Engl J Med 2017;377:2024–35. 10.1056/NEJMoa1709847 29099333 PMC6472479

[9] Middleton PG , Mall MA , Dřevínek P , et al . Elexacaftor-tezacaftor-ivacaftor for cystic fibrosis with a single phe508del allele. N Engl J Med 2019;381:1809–19. 10.1056/NEJMoa1908639 31697873 PMC7282384

[10] Heijerman HGM , McKone EF , Downey DG , et al . Efficacy and safety of the elexacaftor plus tezacaftor plus ivacaftor combination regimen in people with cystic fibrosis homozygous for the F508del mutation: a double-blind, randomised, phase 3 trial. Lancet 2019;394:1940–8. 10.1016/S0140-6736(19)32597-8 31679946 PMC7571408

[11] Van Calster B , Nieboer D , Vergouwe Y , et al . A calibration hierarchy for risk models was defined: from utopia to empirical data. J Clin Epidemiol 2016;74:167–76. 10.1016/j.jclinepi.2015.12.005 26772608

[12] Keogh RH , Tanner K , Simmonds NJ , et al . The changing demography of the cystic fibrosis population: Forecasting future numbers of adults in the UK. Sci Rep 2020;10:10660. 10.1038/s41598-020-67353-3 32606329 PMC7327064

[13] Olivereau L , Nave V , Garcia S , et al . Adherence to lumacaftor-ivacaftor therapy in patients with cystic fibrosis in France. J Cyst Fibros 2020;19:402–6. 10.1016/j.jcf.2019.09.018 31902692

