# Supplementary material for: Projecting the impact of triple CFTR modulator therapy on intravenous antibiotic requirements in cystic fibrosis using patient registry data combined with treatment effects from randomised trials
Source: Thorax. 2021 Sep 23;77(9):873–81. doi: 10.1136/thoraxjnl-2020-216265 (PMC8940731; doi:10.1136/thoraxjnl-2020-216265)
Supplement: Supplementary data [file thoraxjnl-2020-216265supp001.pdf]

Supplementary Materials

Projecting the impact of triple CFTR modulator therapy on intravenous antibiotic requirements in cystic fibrosis using patient registry data combined with treatment effects from randomised trials

Ruth H Keogh, DPhil; Rebecca Cosgriff, MA; Eleni-Rosalina Andrinopoulou, PhD; Keith G Brownlee; Siobhán B Carr, MSc; Karla Diaz-Ordaz, PhD; Emily Granger, PhD; Nicholas P Jewell, PhD; Alex Lewin, PhD; Clemence Leyrat, PhD; Daniela K Schlüter, PhD; Maarten van Smeden, PhD; Rhonda D Szczesniak, PhD; Gary J Connett, MD.

S1. Prediction model specification

The outcomes are counts measured in days and many individuals have a count of zero. To account for this, the analysis uses a hurdle model, which is a two-part model where the first part is a logistic model for the probability of a zero, and the second part is a zero-truncated negative binomial model for positive counts. Let Y_i denote the count outcome for individual i, taking values $0,1,2,\ldots$, and X_i denote the vector of covariates.

The first part of the model is a logistic model for the probability of a zero count:

$$\Pr(Y_i = 0|X_i) = \frac{\exp(\alpha_0 + \alpha^\top X_i)}{1 + \exp(\alpha_0 + \alpha^\top X_i)} \tag{1}$$

The second part of the model is a truncated negative binomial for the positive counts

$$\Pr(Y_i = y|X_i, Y_i > 0) = \frac{\Pr(Y_i = y|X_i)}{1 - \Pr^{NB}(Y_i = 0|X_i)} \tag{2}$$

where, using the negative binomial distribution,

$$\Pr(Y_i = y|X_i) = \frac{\Gamma(y+\theta)}{y!\,\Gamma(\theta)}\left(\frac{\theta}{e^{\beta_0+\beta^\top X_i} + \theta}\right)^{\theta}\left(\frac{e^{\beta_0+\beta^\top X_i}}{e^{\beta_0+\beta^\top X_i} + \theta}\right)^{y} \tag{3}$$

and $\Pr^{NB}(Y_i = 0|X_i)$ denotes the probability of a zero count under the negative binomial model. The parameter θ is the over-dispersion parameter, and $\Gamma(\cdot)$ is the gamma function.

The models were fitted in R (version 3.6.3) using the ‘hurdle’ function from the ‘pscl’ package.[1]

Supplementary Table 4 shows the estimated parameters from fitting the hurdle model for the two outcomes (hospital-IV-days and home-IV-days), and bootstrap standard errors. All models (both parts) include the set of covariates listed in Table 1. Continuous covariates (FEV1%, FEV1% in the previous year, age and BMI) were modelled using restricted cubic splines with knots at the 10th, 50th and 90th percentiles. Count variables (hospital- and home-IV-days over the past year) were modelled using a binary variable indicating a zero and a restricted cubic spline for the non-zero part with knots at the 10th, 50th and 90th percentiles. A restricted cubic spline with three knots results in two terms in the regression, i.e. two parameters to estimate.

For FEV1% (and FEV1% in the previous year) the knots were at 33.6, 68.2, 96.7. For age the knots were at 14.8, 25.6, 44.1 and for BMI they were at 17.9, 21.8, 27.2. For the non-zero part of hospital-IV-days over the past year the knots were at 4, 15, 58, and for the non-zero part of home-IV-days over the past year they were at 7, 20, 53.

S2. Imposing treatment effects and calculating prediction intervals

S2.1 Imposing treatment effects

Approach 1

For Approach 1 the expected impact of TEZ/IVA is imposed on each eligible individual's observed FEV1% value in the 2018 data, and the expected impact of ELEX/TEZ/IVA is then additionally imposed.

The effect of TEZ/IVA is imposed on FEV1% using the results from of Taylor-Cousar et al (2017)[2] as this study was larger than that of Rowe et al (2017)[3]. To take account of the uncertainty in the RCT estimate of the treatment effect, a treatment effect is sampled from its estimated posterior distribution: a normal distribution with mean 3.4 and standard deviation 0.33 (based on the reported 95% CI). This value is added to their observed FEV1%. The modified FEV1% value is then used, along with other covariates, in the prediction models to obtain predictions of hospital-IV-days and home-IV-days for each individual.

We used the results of Middleton et al (2019)[4] to impose the treatment effects for ELEX/TEZ/IVA for F508del heterozygous individuals eligible only for ELEX/TEZ/IVA and the results of Heijerman et al (2019)[5] to impose the treatment effects for ELEX/TEZ/IVA for individuals also eligible for TEZ/IVA. Using the results of Middleton et al (2019)[4] a value for the treatment effect is sampled from a normal distribution with mean 13.6 and standard deviation 0.61 for each eligible individual and added to their observed FEV1%. Using the results of Heijerman et al (2019)[5] a value is sampled from a normal distribution with mean 10.4 and standard deviation 0.91 for each eligible individual and added to their modified FEV1% obtained after imposing the TEZ/IVA effect. The modified FEV1% value is then used, along with other covariates, in the prediction models to obtain predictions of hospital-IV-days and home-IV-days for each individual.

The sampling of the treatment effects is repeated 1000 times and the population total and mean of hospital-IV-days, home-IV-days and combined-IV-days recorded for each sample: the reported results are the average over the 1000 estimates.

Approach 2

For Approach 2 we first used our models to obtain the predicted hospital-IV-days and home-IV-days for each individual. The predicted values for those eligible for TEZ/IVA are then reduced by a factor based on the trial evidence on the impact of the treatment on exacerbation rate. The predicted values for those eligible for ELEX/TEZ/IVA are then reduced further by a factor.

We again used the results of Taylor-Cousar et al (2017)[2] to impose the treatment effects for TEZ/IVA. To take account of the uncertainty in the trial estimate of the rate ratio, a value is sampled from a normal distribution with mean log(0.65) and standard deviation 0.15 for each eligible individual. The predicted hospital-IV-days and home-IV-days are then reduced by a proportion given by the exponential of the sampled value. We use the results of Middleton et al (2019)[4] to impose the treatment effects for ELEX/TEZ/IVA. The study of Heijerman et al (2019)[5] who compared ELEX/TEZ/IVA with TEZ/IVA did not report on exacerbation rates, and therefore we apply the results of Middleton et al (2019)[4] to all individuals eligible for ELEX/TEZ/IVA, including those who are also eligible for TEZ/IVA. A value is sampled from a normal distribution with mean log(0.37) and standard deviation 0.20 for each eligible individual. The predicted hospital-IV-days and home-IV-days are then reduced by a proportion given by the exponential of the sampled value.

S2.2 Prediction intervals

Prediction intervals are different from confidence intervals and are wider than confidence intervals. The 95% prediction interval is the range within which the predicted future observed number of individuals is expected to lie with 95% probability. Prediction intervals take into account variability in the estimated model parameters

and the sampling variability of predicted outcomes. For prediction intervals for the population totals of hospital-IV-days, home-IV-days, and combined-IV-days this includes taking into account the variability in the estimation of the effects of taken from the RCTs. A parametric model-based resampling procedure, which is related to parametric bootstrapping, was used, making use of methods described by Davison and Hinkley (1997, Section 6.3.3)[6] and Mandel (2013)[7].

The hurdle model (equations 1-3) includes 25 parameters in each part of the model (the logistic and zero-truncated negative binomial models) plus the over-dispersion parameter θ from the zero-truncated negative binomial model, giving a total of 51 parameters. The prediction model was fitted on data from 2015-2017, in which many individuals are repeated, and hence we used bootstrapping to estimate the variance-covariance matrix for the model parameters. The parameters were estimated in 1000 bootstrap samples and the empirical variance-covariance matrix of the resulting estimates was obtained (using the log of the overdispersion parameter, $\log\theta$). We denote the combined vector of model parameters by $\boldsymbol{\beta}$ and their estimates by $\widehat{\boldsymbol{\beta}}$ and estimated variance-covariance matrix by Σ .

Below we outline the steps used to obtain 95% prediction intervals under Approaches 1 and 2 (see Figure 1 main article). The procedure is performed separately for the scenarios presented in Tables 3 and 4 in the main text: "No treatment effects", "With TEZ/IVA effect applied to eligible individuals", "With ELEX/TEZ/IVA effect applied to eligible individuals, including those assumed to switch from TEZ/IVA".

Approach 1

1. Take a random draw of the parameters $\boldsymbol{\beta}$ from a multivariate normal distribution with mean $\widehat{\boldsymbol{\beta}}$ and variance-covariance matrix Σ.
2. Take a single random draw of the treatment effect parameter (see Figure 1 main text, Approach 1, and Section S2.1) and modify the 2018 FEV1% data for individuals in the relevant genotype groups for each scenario ("With TEZ/IVA effect applied to eligible individuals", "With ELEX/TEZ/IVA effect applied to eligible individuals, including those assumed to switch from TEZ/IVA"). This gives a modified set of values for the predictor variables for each individual.
3. For each individual take a random draw of the outcome from the hurdle model with parameter draws obtained in step 1 and the modified values for their predictor variables from step 2. This was done using the 'rzanegbin' function from the VGAM package in R.[8]
4. Sum the random outcome draws from step 3 across all individuals to give the population total.
5. Repeat steps 1-4 1000 times, i.e. using different random draws each time in steps 1-3. The lower and upper limits of the 95% prediction interval are calculated as the 0.025 and 0.975 percentiles of the distribution of the 1000 population totals respectively. 95% prediction intervals for the mean outcome (Table 4a) were obtained in the same way after calculating the population totals.

Approach 2

1. Take a random draw of the parameters $\boldsymbol{\beta}$ from a multivariate normal distribution with mean $\widehat{\boldsymbol{\beta}}$ and variance-covariance matrix Σ.
2. For each individual take a random draw of the outcome from the hurdle model with parameter draws obtained in step 1 and the observed values for their predictor variables. This was done using the 'rzanegbin' function from the VGAM package in R.[8]
3. Take a single random draw of the treatment effect parameter (see Figure 1 main article, Approach 2, and Section S2.1). Use this to apply the modifications to the outcome draws from step 2.
4. Sum the modified random outcome draws from step 3 across all individuals to give the population total.

5. Repeat steps 1-4 1000 times, i.e. using different random draws each time in steps 1-3. The lower and upper limits of the 95% prediction interval are calculated as the 0.025 and 0.975 percentiles of the distribution of the 1000 population totals respectively. 95% prediction intervals for the mean outcome (Table 4b) were obtained in the same way after calculating the population totals.

For prediction intervals in the "No treatment effects" scenario, we follow the steps for Approach 1 omitting step 2 (and using observed predictor values in step 3), or equivalently follow the steps for Approach 2 omitting step 3.

Supplementary Table 1: CFTR modular treatments and access in the UK

Treatment (brand name)	Which genotype group effective in (based on RCTs)	Access in the UK
Ivacaftor<br>(Kalydeco)	Gating mutations	• Since 2012/13<br>• Gating mutation (G551D, G178R, S549N, S549R, G551S, G1244E, S1251N, S1255P or G139D), age 6 months+<br>• R117H mutation, age 18+
Ivacaftor+Lumacaftor (IVA/LUMAC)<br>(Orkambi)	2 copies of F508del	• Since 2019, age 2+<br>• https://clinicaltrials.gov/ct2/show/NCT01807923?term=ivacaftor+lumacaftor&cond=cystic+fibrosis&draw=4&rank=28
Tezacaftor+Ivacaftor (TEZ/IVA)<br>(Symkevi)	• 2 copies of F508del<br>• 1 copy of F508del plus a residual function mutation	• Compassionate grounds since Sept 2017 (FEV1%<=40%)<br>• Since 2019, age 12+<br>• Most eligible patients aged 12+ are treated with Symkevi as opposed to Orkambi<br>• https://www.clinicaltrials.gov/ct2/show/NCT03278314?term=expanded+access&cond=Cystic+Fibrosis&draw=2&rank=2
Elexacaftor+Ivacaftor+Tezacaftor<br>(ELEX/TEZ/IVA) (Kaftrio)	• 2 copies of F508del<br>• 1 copy of F508del plus a minimal function mutation	• Compassionate grounds since August 2019 (FEV1%<=40%)<br>• Approved in the UK June 2020

Supplementary Table 2: Summary of trial evidence on TEZ/IVA (Symkevi) and ELEX/TEZ/IVA (Kaftrio).

	N	Comparator	Main eligibility criteria	Primary outcome	Secondary outcomes
**TEZ/IVA**					
Taylor-Cousar et al. (2017)	510	Placebo	F508del homozygous<br>Aged >12<br>FEV1% 40%-90%<br>Stable disease as judged by the investigators	Increase in FEV1% at 24 weeks: 4.0 (95% CI 3.1-4.8)	Rate of pulmonary exacerbations that led to hospitalization or treatment with intravenous antibiotic agents (or both) up to week 24 (annualised): 0.65 (95% CI 0.48-0.88)
Rowe et al. (2017)	248	Placebo	F508del homozygous or F508del heterozygous with a residual function mutation<br>Aged >12<br>FEV1% 40%-90%	Increase in FEV1% at 4 weeks and 8 week (average): 6.8 (95% CI 5.7-7.8)	Exacerbation rate over 8 weeks (reported as an 'exploratory endpoint' rather than a secondary endpoint): 0.54 (95% CI 0.26,1.13)
**ELEX/TEZ/IVA**					
Middleton et al. (2019)	403	Placebo	F508del heterozygous with a minimal function mutation<br>Aged >12<br>FEV1% 40%-90%<br>Stable disease during the 28-day screening period before the first dose of active treatment or placebo	Change in FEV1% at 4 weeks: 13.8 (95% CI 12.1-15.4)	Exacerbations up to 24 weeks post-baseline, and the exacerbation rate was estimated to be reduced by 63% (Rate ratio 0.37, 95% CI 0.25,0.55). This result refers to all exacerbations. They also separately investigated exacerbations leading to hospitalisation (Rate ratio 0.29, 95% CI 0.14-0.61) and exacerbations leading to treatment with IV antibiotics (Rate ratio 0.22, 95% CI 0.11-0.43).
Heijerman et al. (2019)	113	TEZ/IVA	F508del homozygous<br>Aged >12<br>FEV1% 40%-90%<br>stable cystic fibrosis as judged by the investigators	Change in FEV1% at 4 weeks: 10.0 (95% CI 7.4-12.6)	Change from baseline at week 4 in sweat chloride concentration and in the Cystic Fibrosis Questionnaire-Revised respiratory domain

Supplementary Figure 1: Plots showing the distribution of the non-zero values for the outcomes hospital-IV-days and home-IV-days, using data from 2015-2017. As noted in the main text we considered extended hurdle models allowing additional peaks but these did not provide materially improved predictions. Our focus, however, was on estimating the mean counts and population totals. If our aim were to estimate other features of the distribution, then more complex models may provide improved predictions.

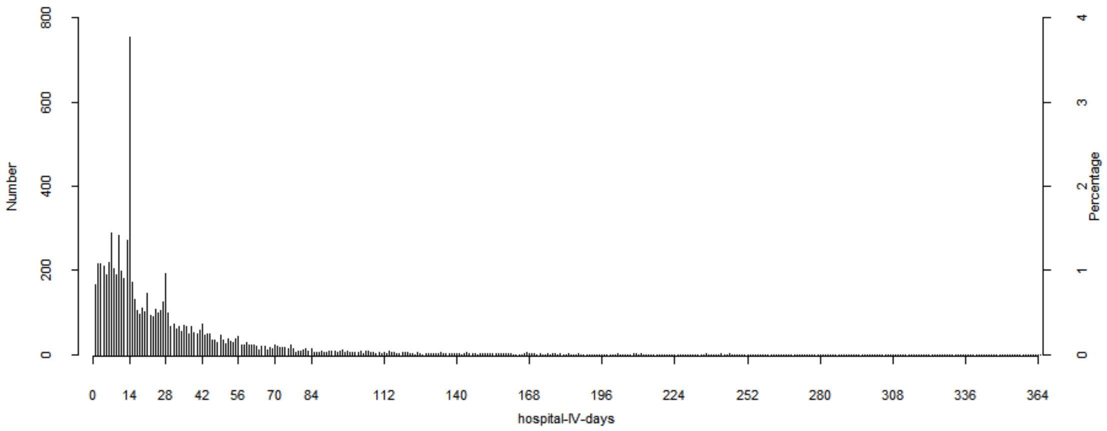

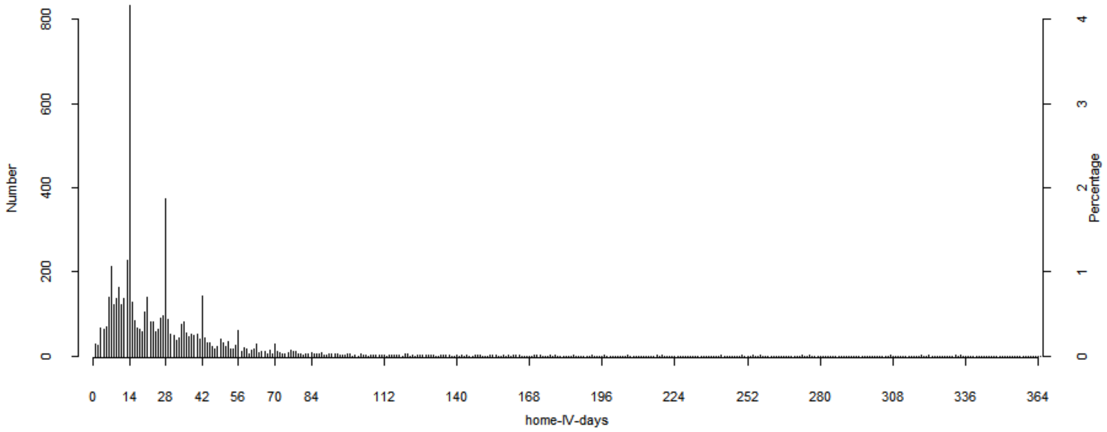

Supplementary Table 3: Descriptive statistics for covariates and outcomes, including missing data, by year. This table summarises covariates and outcomes for a total of 7461 individuals aged over 12 who had data recorded in the Registry at least once between 2015 and 2018, after excluding individuals post-transplant. After using the last observation carried forward for missing covariates and excluding any remaining rows with missing covariate data (where there was no previous value to carry forward), there remain 18744 annual data records on 6962 individuals for 2015-2017, and 6407 individuals in 2018. After further excluding 777 individuals (4.1%) with missing outcomes for 2015-2017 there remain 17967 annual data records on 6731 individuals for 2015-2017.

		2015 (n=6215)	2016 (n=6377)	2017 (n=6524)	2018 (n=6499)
**Covariates**					
Age	Median (IQR)	25.2 (19.0,54.0)	25.9 (19.0,54.7)	26.1 (19.3,55.7)	26.5 (19.5,56.2)
Sex	Male n (%)	3337 (53.7%)	3429 (53.8%)	3520 (54.0%)	3485 (53.6%)
	Female n (%)	2878 (46.3%)	2948 (46.2%)	3004 (46.0%)	3014 (46.4%)
FEV1%	Median (IQR)	67.7 (48.1,84.9)	68.7 (48.8,84.9)	68.2 (48.1,85.5)	69.0 (48.3,85.7)
	Missing n (%)	271 (4.4%)	327 (5.1%)	249 (3.8%)	161 (2.5%)
FEV1% previous year	Median (IQR)	69.2 (49.8,85.8)	69.7 (50.4,86.1)	70.2 (50.8,86.0)	70.2 (50.2,86.3)
	Missing n (%)	333 (5.4%)	325 (5.1%)	387 (5.9%)	241 (3.7%)
Body mass index	Median (IQR)	21.8 (19.5,24.3)	21.8 (19.5,24.3)	21.8 (19.6,24.5)	21.8 (19.6,24.4)
	Missing n (%)	131 (2.1%)	126 (2.0%)	125 (1.9%)	35 (0.5%)
P.aeruginosa	No n (%)	2538 (40.8%)	2822 (44.3%)	2939 (45.0%)	2973 (45.7%)
	Yes n (%)	3677 (59.2%)	3446 (54.0%)	3480 (53.3%)	3522 (54.2%)
	Missing n (%)	0 (0.0%)	109 (1.7%)	105 (1.6%)	4 (0.1%)
S. aureus	No n (%)	3639 (58.6%)	3967 (62.2%)	3947 (60.5%)	3945 (60.7%)
	Yes n (%)	2576 (41.4%)	2301 (36.1%)	2472 (37.9%)	2550 (39.2%)
	Missing n (%)	0 (0.0%)	109 (1.7%)	105 (1.6%)	4 (0.1%)
B. cepacia	No n (%)	5911 (95.1%)	5955 (93.4%)	6121 (93.8%)	6189 (95.2%)
	Yes n (%)	304 (4.9%)	313 (4.9%)	298 (4.6%)	306 (4.7%)
	Missing n (%)	0 (0.0%)	109 (1.7%)	105 (1.6%)	4 (0.1%)
CF-related diabetes	No n (%)	3876 (62.4%)	3971 (62.3%)	4068 (62.4%)	4172 (64.2%)
	Yes n (%)	2339 (37.6%)	2406 (37.7%)	2456 (37.6%)	2327 (35.8%)
Genotype	F508del Homozygous	3050 (49.1%)	3097 (48.6%)	3137 (48.1%)	3151 (48.5%)
	F508+minimal	1103 (17.7%)	1123 (17.6%)	1129 (17.3%)	1117 (17.2%)
	F508+residual	322 (5.2%)	337 (5.3%)	359 (5.5%)	369 (5.7%)
	F508+other/unknown	554 (8.9%)	570 (8.9%)	588 (9.0%)	576 (8.9%)
	Any gating mutation or R117H	710 (11.4%)	740 (11.6%)	786 (12.0%)	774 (11.9%)
	Other/unknown	476 (7.7%)	510 (8.0%)	525 (8.0%)	512 (7.9%)
Hospital-IV-days, past year*	Zero n (%)	3044 (49.0%)	3845 (60.3%)	3813 (58.4%)	3763 (57.9%)
	Median (IQR)	25.0 (14.0,42.0)	15.0 (9.0,32.0)	14.0 (9.0,31.0)	15.0 (9.0,32.0)
	Missing n (%)	99 (1.6%)	84 (1.3%)	124 (1.9%)	76 (1.2%)
Home-IV-days, past year*	Zero n (%)	3040 (48.9%)	4418 (69.3%)	4518 (69.3%)	4505 (69.3%)
	Median (IQR)	26.0 (14.0,43.0)	18.0 (13.0,33.0)	20.0 (13.0,34.0)	19.0 (13.0,34.0)
	Missing n (%)	99 (1.6%)	84 (1.3%)	124 (1.9%)	76 (1.2%)
**Outcome variables**					
Hospital-IV-days*,**	Zero n (%)	3653 (58.8%)	3635 (57.0%)	3485 (53.4%)	-
	Median (IQR)	15.0 (9.0,33.0)	15.0 (9.0,33.0)	16.0 (9.5,33.0)	-
	Missing n (%)	172 (2.8%)	206 (3.2%)	432 (6.6%)	-
Home-IV-days*,**	Zero n (%)	4183 (67.3%)	4304 (67.5%)	4207 (64.5%)	-
	Median (IQR)	18.0 (13.0,34.0)	20.0 (13.0,34.0)	20.0 (13.0,35.0)	-
	Missing n (%)	172 (2.8%)	206 (3.2%)	432 (6.6%)	-

***** When the start and end dates for a given episode were the same, the number of days was counted as 1, otherwise the number of days for that episode was counted as the difference between the start and end dates.

** These are the counts of hospital-IV-days and home-IV-days in the year following the annual review visit in 2015, 2016, 2017. By contrast 'Hospital IV days, past year' and 'Home IV days, past year' are the counts in the year leading up to the annual review visit.

Supplementary Table 4: Estimates (Est) of parameters in the prediction models for hospital-IV-days and home-IV-days, and standard errors (SE) obtained using bootstrapping with 1000 bootstrap samples. Continuous variables were modelled using restricted cubic splines and contribute two terms to the models – see Section S1.

	**Hospital-IV-days**				**Home-IV-days**			
	Model for zero		Model for non-zero counts		Model for zero		Model for non-zero counts	
	Est	SE	Est	SE	Est	SE	Est	SE
Intercept	3.02	0.281	3.911	0.141	-1.411	0.314	3.23	0.142
FEV1%: term 1	-0.035	0.004	-0.016	0.002	-0.017	0.004	-0.007	0.002
FEV1%: term 2	0.004	0.004	0.006	0.002	0.003	0.005	0.004	0.002
FEV1% previous year: term 1	0.013	0.004	0.002	0.002	0.005	0.004	-0.001	0.002
FEV1% previous year: term 2	-0.008	0.004	0.0004	0.002	-0.01	0.004	0.001	0.002
F508+minimal	-0.008	0.048	0.013	0.026	-0.054	0.05	-0.03	0.025
F508+residual	-0.171	0.088	-0.126	0.051	-0.362	0.098	-0.03	0.056
F508+other/unknown	-0.231	0.071	0.044	0.039	-0.204	0.077	-0.015	0.04
Any gating mutation or R117H	-0.454	0.066	-0.085	0.041	-0.621	0.078	-0.072	0.038
Other/unknown	-0.158	0.072	-0.083	0.04	-0.369	0.083	-0.114	0.038
Age: term 1	-0.037	0.005	-0.015	0.003	0.026	0.006	-0.002	0.003
Age: term 2	0.024	0.007	0.017	0.004	-0.039	0.007	0	0.004
Sex: female	0.247	0.036	0.02	0.02	0.411	0.039	0.083	0.019
CF-related diabetes	0.257	0.041	0.098	0.021	0.108	0.042	0.02	0.019
Body mass index: term 1	-0.075	0.013	-0.023	0.007	0.05	0.015	-0.001	0.007
Body mass index: term 2	0.063	0.014	0.012	0.008	-0.068	0.016	0.001	0.008
Hospital-IV-days, past year: zero	-0.615	0.08	0.184	0.048	0.046	0.085	-0.005	0.032
Hospital-IV-days, past year: non zero, term 1	0.054	0.005	0.033	0.002	-0.019	0.005	-0.001	0.002
Hospital-IV-days, past year: non zero, term 2	-0.082	0.011	-0.042	0.005	0.022	0.01	0.005	0.004
Home-IV-days, past year: zero	0.243	0.095	0.117	0.048	-1.037	0.098	0.089	0.044
Home-IV-days, past year: non zero, term 1	-0.001	0.005	-0.001	0.003	0.058	0.005	0.022	0.002
Home-IV-days, past year: non zero, term 2	-0.0001	0.007	-0.0004	0.004	-0.066	0.007	-0.015	0.003
P.aeruginosa	0.308	0.039	0.081	0.023	0.28	0.044	0.053	0.022
S. aureus	0.108	0.037	0.007	0.02	-0.047	0.038	0.012	0.02
B. cepacia	0.214	0.081	-0.052	0.042	0.282	0.089	0.076	0.042
Over-dispersion parameter θ (log scale)	-	-	0.585	0.022	-	-	1.028	0.025

References

1. Jackman S. pscl: Political Science Computational Laboratory. R package version 1.5.5. 2020. https://CRAN.R-project.org/package=pscl

2. Taylor-Cousar, Munck A, McKone EF, et al. Tezacaftor–Ivacaftor in Patients with Cystic Fibrosis Homozygous for Phe508del. *N Engl J Med* 2017; **377**: 2013-2023.

3. Rowe SM, Daines C, Ringshausen FC, et al. Tezacaftor–Ivacaftor in Residual-Function Heterozygotes with Cystic Fibrosis. N Engl J Med 2017; **377**:2024-2035.

4. Middleton PG, Mall MA, Dřevínek P, et al. Elexacaftor-tezacaftor-ivacaftor for cystic fibrosis with a single phe508del allele. *N Engl J Med* 2019; **381**: 1809-19.4.

5. Heijerman HGM, McKone EF, Downey DG, et al. Efficacy and safety of the elexacaftor plus tezacaftor plus ivacaftor combination regimen in people with cystic fibrosis homozygous for the F508del mutation: a double-blind, randomised, phase 3 trial. *Lancet* 2019. doi:10.1016/S0140-6736(19)32597-8.

6. Davison AC, and Hinkley DV. Bootstrap methods and their application. (Cambridge University Press, 1997).

7. Mandel M. Simulation-based confidence intervals for functions with complicated derivatives. *The American Statistician* **67,** 76-81 (2013).

8. Yee T. VGAM: Vector Generalized Linear and Additive Models. R package version 1.1-3. 2020. https://CRAN.R-project.org/package=VGAM
